# Response to letter to the editor: "Mobilization with movement is effective in patients operated for distal radius fracture"

**DOI:** 10.1590/1806-9282.20251811

**Published:** 2026-06-29

**Authors:** Levent Horoz, Basak Cigdem-Karacay, Ismail Ceylan, Halil Alkan

**Affiliations:** 1 Kirsehir Ahi Evran University, Faculty of Medicine, Department of Orthopedics and Traumatology – Kırşehir, Turkey.; 2 Kirsehir Ahi Evran University, Faculty of Medicine, Department of Physical Medicine and Rehabilitation – Kırşehir, Turkey.; 3 Kırsehir Ahi Evran University, School of Physiotherapy and Rehabilitation – Kırşehir, Turkey.; 4 Mus Alparslan University, Faculty of Health Science, Department of Physiotherapy and Rehabilitation – Muş, Turkey.

Dear Editor,

In recent years, the effectiveness of manual therapy applications in patients operated on for distal radius fractures has been investigated^1^. The primary mechanism of action of one such manual therapy method, Mobilization with Movement (MWM), is to improve biomechanical function by correcting joint malalignment. Therefore, Wan and Li^2^ are correct in their view on the importance of joint alignment after distal radius fracture. In this regard, while planning our study, we were meticulous about including information regarding joint alignment after distal radius fracture surgery in the research article. We added the patients’ fracture reduction parameters—volar tilt and radial inclination degrees, radial height, and ulnar variance values—to the study by examining their postoperative radiographs. In our study, no statistical difference was found between these measurements in the control group and the MVM group^3^. The number of studies evaluating the effectiveness of MVM in patients undergoing distal radius fracture surgery is quite limited in the literature. In the study by Tomruk et al. evaluating the effectiveness of MVM in patients undergoing distal radius fracture surgery, the radial inclination, radial height, and volar inclination angles of fracture reduction parameters are included in a table. In the study by Tomruk et al., no difference was found between the intervention group and the control group in terms of fracture reduction parameters^4^.

Unfortunately, the sample size of our study was not sufficient to examine the response to MVM treatment by grouping patients according to fracture reduction parameters. However, we support the hypothesis that the effectiveness of the MWM method will differ between groups when patients are grouped according to these parameters. Future research focusing on the effectiveness of MVM according to fracture reduction parameters in this patient population will contribute to the literature.

## Data Availability

The datasets generated and/or analyzed during the current study are available from the corresponding author upon reasonable request.
